# Case report of a rare postoperative cervical lymph node metastasis of palate mucoepidermoid carcinoma

**DOI:** 10.3389/fonc.2026.1878703

**Published:** 2026-07-30

**Authors:** XianYi Gan, Yan Huang, Tao Zhang, Tao Huang, WeiQuan Xu, ChunPing Liu, Shuai Li

**Affiliations:** 1 Department of Oral and Maxillofacial Surgery, College & Hospital of Stomatology, Guangxi Medical University, Nanning, Guangxi, China; 2 Guangxi Clinical Research Center for Craniofacial Deformity, Nanning, Guangxi, China; 3 Guangxi Key Laboratory of Oral and Maxillofacial Rehabilitation and Reconstruction, Nanning, Guangxi, China

**Keywords:** case report, follow-up, mucoepidermoid carcinoma, neck metastasis, surgical resection

## Abstract

**Objective:**

This study aims to investigate the clinicopathological characteristics, imaging features, diagnostic approaches, and treatment strategies of Mucoepidermoid Carcinoma (MEC).

**Methods:**

We performed a retrospective analysis of the clinical data of a patient admitted to the department of oral and maxillofacial surgery at College of Stomatology, GuangXi Medical University in 2025. Surgery was performed for inpatient treatment, during which the right cervical tumor was completely removed and cervical lymph node dissection was completed, and a 11-month postoperative follow-up.

**Results:**

This case describes a 47-year-old Asian female patient who developed tumor metastasis to the right cervical region 11 years after undergoing resection of a well-differentiated MEC in the right palate. Physical examination revealed multiple nodular and mass-like lymph nodes in the right neck. Postoperative histopathological examination confirmed the diagnosis of MEC.

**Conclusion:**

This study focused on describing the clinical characteristics of a case with MEC, highlighting that although the initial treatment was effective, the risk of neck metastasis persists. The unique insights gained from this case help us better understand the long-term development pattern of MEC, emphasize the importance of long-term close follow-up, and highlight postoperative radiotherapy.

## 1 Introduction

MEC is recognized as one of the most prevalent malignant tumors of the salivary glands, accounting for approximately 10-15% of all salivary gland neoplasms and representing 30% of all malignant salivary gland tumors (1), with a higher prevalence observed among females, particularly those around the middle-aged (2). Current therapeutic strategies for MEC primarily focus on surgical interventions, with the aim of achieving complete tumor resection (3). A low cervical lymph node metastasis rate of approximately 10.6% has been reported for MEC (4), with lymph node involvement being relatively rare observed even after prolonged follow-up (5).

This demographic highlights the substantial health burden imposed by MEC on both patients and the healthcare system. Moreover, the management of MEC is challenged by its potential for local recurrence and metastasis, underscoring the need for a comprehensive understanding of its epidemiology and pathophysiology. The present case illustrates the complexities associated with MEC management and provides a basis for investigating the underlying mechanisms of cervical lymph node metastasis. This case report describes a MEC patient’s cervical lymph node metastasis, adding to the literature and providing a reference for similar cases. The report was prepared following the CARE guidelines checklist.

## 2 Case presentation

### 2.1 General information

In 2013, a 35- year- old female was diagnosed with right palatal MEC and underwent expansion resection of the right maxilla and free transplantation of an abdominal flap. Postoperative recovery was uneventful, and regular follow-ups were conducted without abnormalities. In 2023, a gradually enlarging mass in the right upper cervical region, approximately the size of a quail egg, without discomfort. Then, the lesion gradually increased. While it has grown more rapidly in 2024. There was no obvious pain upon palpation.

### 2.2 Specialized information

**Figure 1:**
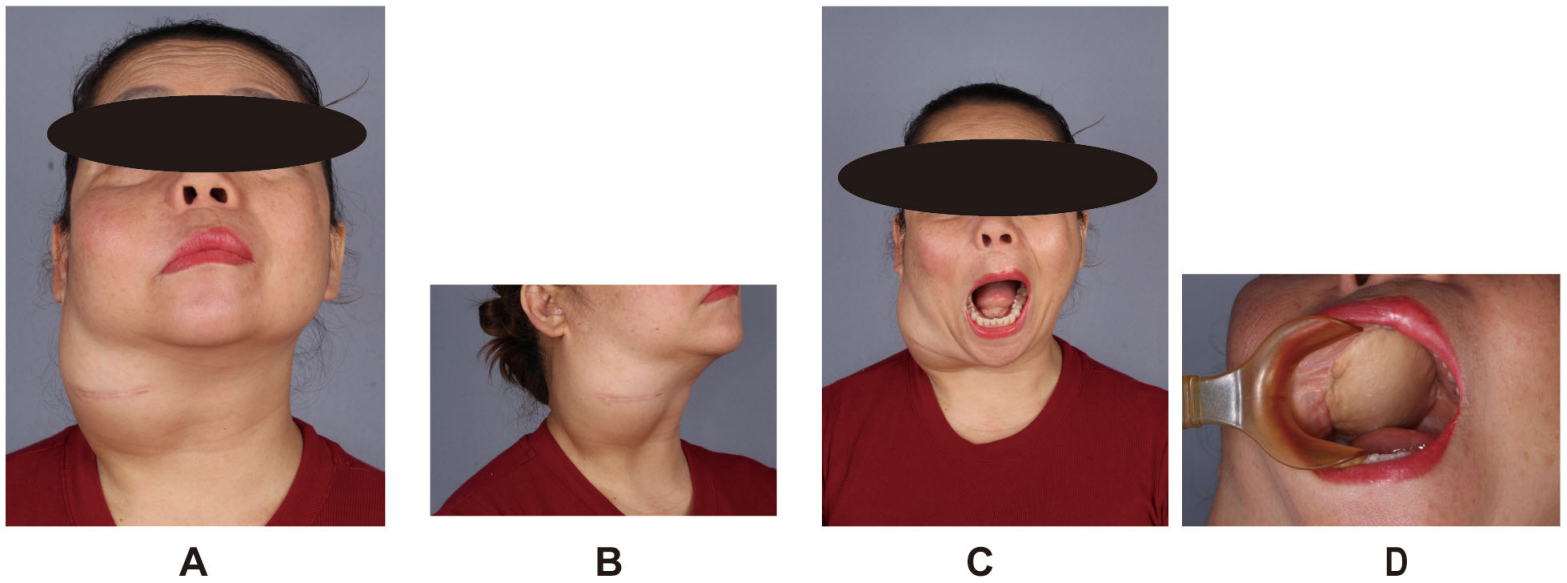
A large space-occupying lesion in the right cervical region of the patient. **(A)**. Space-occupying lesion visible on the right side of the neck, **(B)**. Cervical scar, **(C)**. Mouth opening (or range of mouth opening), **(D)**. Intraoral findings.

A mass was observed in the right upper cervical region, measuring approximately 8.0 cm × 7.0 cm × 6.0 cm. On palpation, the mass had an indistinct border, a relatively firm texture, and poor mobility, with adherence to the surrounding tissues (**Figure 1A**). The overlying skin showed the original surgical scar, approximately 12 cm in length (**Figure 1B**). Mouth opening was normal (**Figure 1C**). On the right side of the oral cavity, the original transplanted flap appeared normal in color, with no ulceration or neoplasm (**Figure 1D**). No palpable enlarged lymph node was detected in the left neck. Further details are shown in **Figure 1**.

### 2.3 Supplementary examinations

#### 2.3.1 Physical examination

**Figure 2:**
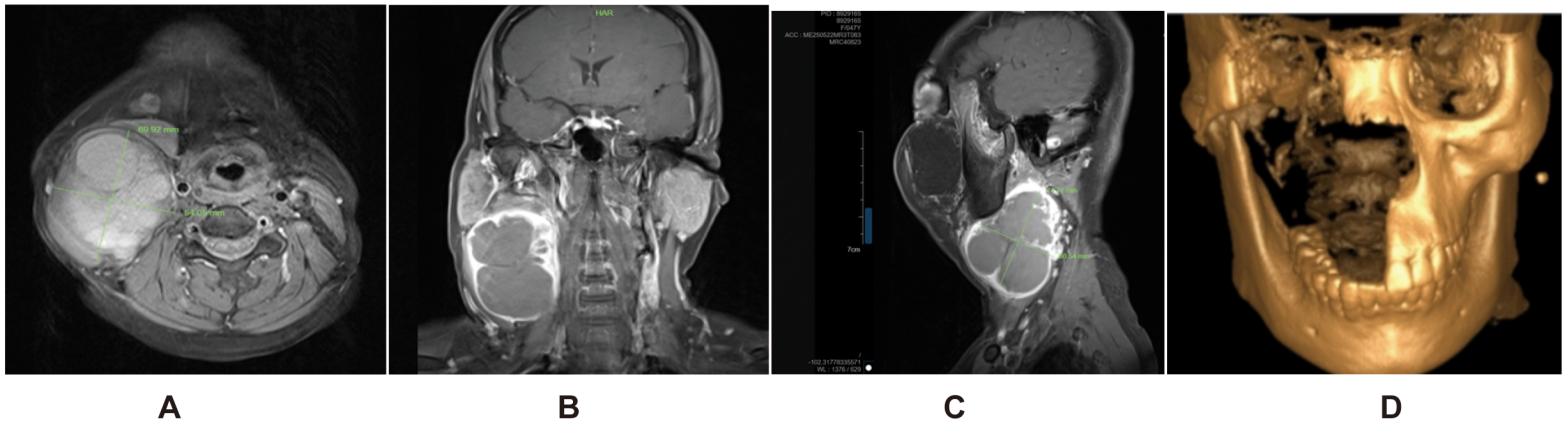
The disease of the patient as depicted on the MRI and CT scans. **(A)**. Axial MRI, **(B)**. Coronal MRI, **(C)**. Sagittal MRI, **(D)**. CBCT reconstruction.

Magnetic resonance imaging (MRI) (May 22, 2025) revealed partial bone defect of the right maxilla. No abnormal signal was observed in the left submandibular glands or bilateral parotid glands. Multiple nodular and mass-like enlarged lymph nodes were detected in the right neck, measuring approximately 6.8 cm × 4.9 cm × 8.0 cm (**Figures 2A-C**). The right submandibular gland was compressed, and the right carotid artery and jugular vein were slightly compressed and displaced medially (**Figure 2A**).

Cone-beam computed tomography (CBCT) performed (May 22, 2025) showed defects involving the right maxillary tuberosity, the maxilla, hard palate, inferior orbital wall, zygomatic bone, root of the sphenoid bone, and parts of the inner and outer cortical plates, with soft tissue filling the defects (**Figure 2D**).

Pulmonary CT (May 22, 2025) demonstrated a few fibrotic foci in the lingular segment of the left upper lobe and the medial segment of the right middle lobe. Solid nodules were present in the posterior segment and the lateral basal segment of the right lower lobe, which were considered benign.

### 2.4 Pathology examination

Preoperative pathology for the initial surgery (May 8, 2013): well-differentiated MEC of the right palate. Postoperative pathology following the initial surgery (May 21, 2013): well-differentiated MEC of the right maxilla, with invasion of the inferior turbinate and sphenoid sinus. No malignancy was detected in the nasal mucosa, the posterior margin, or the proximal margin of the tumor.

#### 2.4.1 Treatment and results

Multidisciplinary consultation (6): We held a consultation with the pathology department, the radiology department, and the oncology department. combined the patient’s medical history with imaging examinations, the tumor was identified as a metastatic lesion originating from the primary tumor in the maxilla.

**Figure 3:**
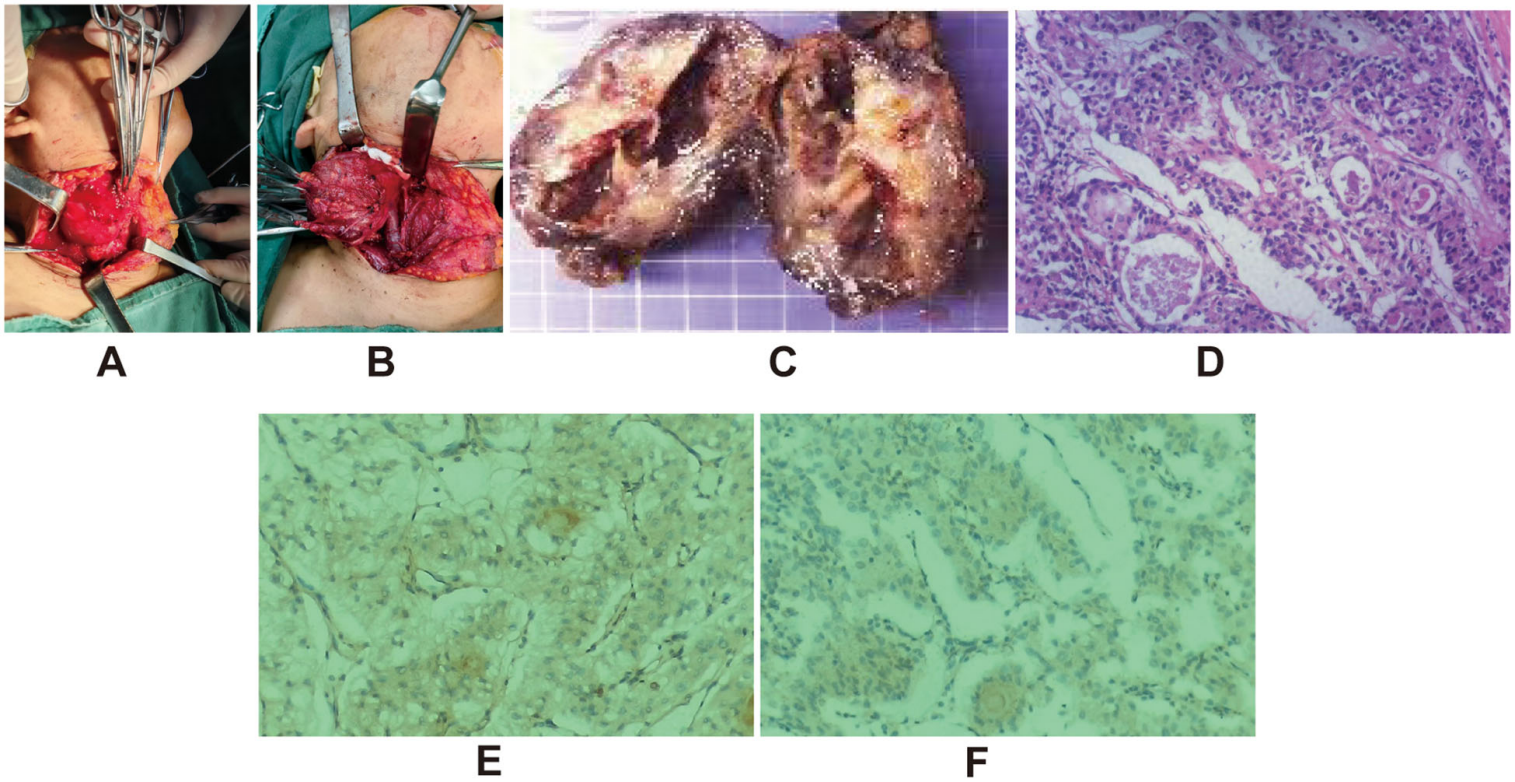
The intraoperative situation and the postoperative pathological results. **(A)**. Relationship with surrounding tissues, **(B)**. Complete separation, **(C)**. Gross appearance of the specimen, **(D)**. H&E staining of the specimen, **(E)**. CK staining of the specimen, **(F)**. p63 staining of the specimen.

Under general anesthesia, a huge mass was observed in the right cervical area, with a complete capsule and adhesion to the surrounding tissues (**Figure 3A**). After blunt dissection, the mass could be largely separated intact (**Figure 3B**). The right cervical I-V area lymph node dissection were performed.

### 2.5 Gross specimen

#### 2.5.1 Pathological results

The tumor has a cystic cavity-like structure and intracystic solid masses (**Figures 3C, D**). The cystic cavity is mostly lined with mucinous cells, accompanied by intermediate cells or epidermoid cells, and there is mucus in the cavity. The interstitium shows inflammatory reaction; these changes combined with the clinical history are consistent with MEC (highly differentiated type). Eleven lymph nodes (levels I–V) in the right neck were identified, with no evidence of positive lymph node status. CK7(**Figure 3E**) and P63 (**Figure 3F**) are positive staining in scattered intermediate cells.

### 2.6 Postoperative follow-up

**Figure 4:**
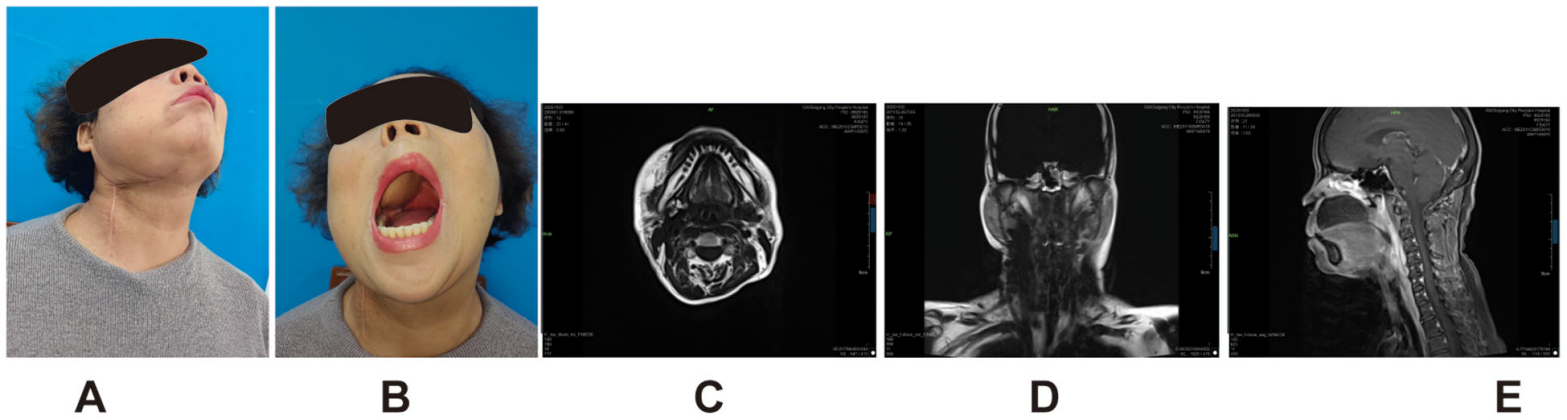
The patient’s follow-up six months after the surgery and the MRI imaging results of the patient 6 months after the surgery. **(A)**. Postoperative view of the neck, **(B)**. Postoperative view of the oral cavity, **(C)**. Postoperative axial MRI, **(D)**. Postoperative coronal MRI, **(E)**. Postoperative sagittal MRI.

The patient recovered well postoperatively. One month after surgery (June 27, 2025), whole-body bone SPECT/CT revealed no evidence of metastasis. Pulmonary CT performed six months postoperatively (November 3, 2025) showed findings almost identical to the preoperative pulmonary CT (May 22, 2025). At the six-month follow-up examination, palpation of the right neck revealed soft tissue without enlarged lymph node (**Figure 4A**). No oral changes or other symptoms of discomfort were reported (**Figure 4B**). Postoperative changes in the neck were observed on magnetic resonance imaging, with no identifiable neoplasm (**Figures 4C-E**). Long-term follow-up of this patient is ongoing.

## 3 Discussion

MEC is a prevalent salivary gland malignancy, accounting for 10–15% of all salivary neoplasms and 30% of malignant cases, with a higher incidence in middle-aged females. Cervical lymph node metastasis is relatively uncommon and is rarely observed even after extended follow-up.

The tumor in the upper cervical region has a cystic cavity-like structure and intracystic solid masses. The cystic cavity is mostly lined with mucinous cells, accompanied by intermediate cells or epidermoid cells, and there is mucus in the cavity. These changes combined with the clinical history are consistent with MEC. Therefore, this entity is highly unlikely to be a recurrence of MEC. It is more consistent with MEC metastasis (4).

Yujiao Li et al. showed higher T grades or N grades would increase the risk of distant metastasis and regional disease progression (7). Stefan Grasl et al. conducted a follow-up study on 212 patients with parotid MEC. In T1/T2 type tumors, the incidence of metastatic lymph nodes was 18.8% (n = 30), while in T3/4 type tumors it was 56.5% (n = 26). The proportion of patients with positive pathological lymph nodes increased with the increase in tumor grade (8). Both surgical pathology reports showed a low-grade pathological grade for this case in 2013. The occurrence of metastasis more than 10 years after the surgery was extremely rare. Moreover, the lesion progressed rapidly, with the cervical mass expanding considerably within just over 3 months.

MEC can spread via lymph node metastasis (9) and iatrogenic dissemination (10). For this case, no tumor recurrence was found in the primary lesion area, but only metastasis occurred in the neck 11 years after the surgery. This phenomenon may suggest a more indolent biological behavior, wherein the tumor’s histological subtype, such as high-grade versus low-grade tumors, plays a critical role in dictating the metastatic profile (11). Recent studies have identified molecular markers, such as MAML2 rearrangements, which are prevalent in MEC and can signify a tumor phenotype, thus warranting closer surveillance post-surgery (12).These findings resonate that high-grade MECs often present with aggressive local behavior yet may exhibit variable patterns of lymphatic spread, potentially due to differences in tumor microenvironment and immune response mechanisms (13).

Radiation therapy is an effective approach to enforce outcomes for MEC (14), and postoperative spatially fractionated radiation therapy (SFRT) is one of the commonly used modalities for reducing local recurrence and regional metastasis, which is an innovative radiotherapy technique characterized by a highly uneven dose distribution. By creating a dose pattern of “high-dose peaks” and “low-dose valleys” within the target volume, SFRT achieves precise targeting of the tumor and effective protection of normal tissues, significantly improving the therapeutic index of radiotherapy. It is highly effective for large and recurrent tumors (15). Therefore, postoperative radiotherapy is recommended for this patient to eradicate residual tumor following gross tumor resection.

This study has several limitations. First, the single-case design limits the generalizability of our findings to diverse MEC populations. Second, the longtime interval since initial surgery caused loss of some treatment data, preventing adequate comparison. Third, fine needle aspiration cytology was not performed due to carotid proximity, cysticity, and patient refusal.

## 4 Conclusion

This case report describes a patient with MEC who developed cervical metastasis 11 years after initial treatment, demonstrating that the risk of regional recurrence persists over an extended period. This case underscores the importance of long-term surveillance and suggests a potential role for radiotherapy in reducing this delayed metastatic risk.

## Data Availability

The raw data supporting the conclusions of this article will be made available by the authors, without undue reservation.
